# Comparison and association of performance indicators according to set outcome and set score difference in AVP women's beach volleyball

**DOI:** 10.3389/fspor.2025.1584173

**Published:** 2025-08-11

**Authors:** Yago Pessoa da Costa, Filipe Santana Martins, Lucas Roberto Ribeiro, Antonio García-de-Alcaraz, Gilmário Ricarte Batista

**Affiliations:** ^1^ Department of Physical Education, Federal University of Paraiba, João Pessoa, Brazil; ^2^ Department of Physical Education, Maurício de Nassau University Center, João Pessoa, Brazil; ^3^ SPORT Research Group (CTS-1024), CIBIS (Centro de Investigación para el Bienestar y la Inclusión Social) Research Center, University of Almería, Almería, Spain

**Keywords:** game actions, performance coefficient, sand sports, notational analysis, team performance, key performance indicators

## Abstract

**Purpose:**

This study aimed to analyze the association between technical-tactical performance indicators and the set outcome, game phase, and set score difference.

**Methods:**

In total, 41 matches, 79 sets, and 14,959 game actions [serve: 2,879; serve reception: 2,567; set: 2,176; attack (side-out): 2,324; block: 818; dig: 1,684; set (counterattack): 1,224; and counterattack: 1,287] from two women’s Association of Volleyball Professionals Gold Series tournaments were analyzed. The independent variables were set outcome (i.e., winner or loser) and set score difference, whereas the dependent variables were points scored in each game phase, performance coefficient, and efficiency. A two-way analysis of variance was employed for comparison purposes and logistic regression was used to analyze the association between the match outcomes and the performance indicators.

**Results:**

Winners scored more points in the K0, K2, and K3B game phases compared to losers. Similarly, higher performance coefficients and efficiencies were observed for actions performed during the defensive phase (block, dig, set, and counterattack). Moreover, the performance during the K2 and K3B phases, attack and counterattack efficiency, and the block and dig performance coefficients were associated with winning the set. The set score difference was characterized as an indicator of set balance because the differences in performance indicators between the winners and losers generally increased with greater point differentials.

**Conclusion:**

In the context of elite women's beach volleyball, although attacking was important for winning a set, the key performance indicators were mainly derived from the construction of counterattacks. In addition, the set score difference reflects the balance of the set. Therefore, these parameters can be used to guide training programs and assess team performance.

## 1 Introduction

Beach volleyball is one of the most popular Olympic sports in the world, with players competing in numerous events worldwide. The Association of Volleyball Professionals (AVP) organizes tournaments around the United States for athletes of different performance levels [i.e., athlete tiers (1)], of which the “Gold Series” events comprise the most elite athletes. In general terms, teams comprise two players, who try to score 21 points, with a difference of two or more points (i.e., 2-0) to win the set (15 points in the third set). During a rally, players exert intense effort to perform jumps and quick movements on the sand and recover in the intervals between rallies (2–4). However, the match outcome is mainly determined by technical-tactical performance. In this context, match analysis is a valuable tool for identifying key performance indicators.

Using this approach, beach volleyball match analysis commonly focuses on the set rather than the match as a whole (5–6). This leads to a more accurate analysis because the score from the first set is not carried over to the next (7). In each set, each point is played in a rally (i.e., the period between the serve and the ball going out of play), and some game phases are characterized according to ball possession. Thus, K0 denotes a serve; K1 (side-out) denotes a serve reception, a set, and an attack; and K2 denotes a block, a dig (i.e., the action of defending an attack), a set, and a counterattack. In addition, depending on whether the ball remains in play after the previous phases, K3A (consisting of a counterattack by the team that performed actions in K1) and K3B (consisting of a counterattack by the team that performed actions in K2) can also occur [adapted from (6, 8)]. The consideration of specific skills related to beach volleyball fundamentals and game phases allows for the identification of technical-tactical performance indicators that can help coaches and players achieve better game performance and understanding.

Previously, performance indicators have been investigated as a function of the set outcome in beach volleyball (6, 9). These indicators can be studied in absolute terms (e.g., the number of points scored) or in standardized terms [e.g., attacking efficiency (10)]. In this sense, the insights provided by Medeiros et al. (6) suggest that the coefficient of performance and counterattack are the key to winning a set at the U19, U-21, and senior levels. However, the competitiveness of a set, in terms of teams playing at an equal level, was not considered by the authors in this study. One way of quantifying the set balance is to examine the point difference between the winning and losing teams (9). Recently, Giatsis et al. (9) reported that attack percentage was a relevant predictor of winning a set, and serve points were also associated with set victories. Although this study provides robust insights, some limitations should be highlighted. The authors did not consider specific beach volleyball game phases (e.g., K0 or K1), and a performance coefficient (i.e., the average score adjusted for efficiency of actions) was not calculated, which could provide valuable information about the relationship between all actions performed within each fundamentals-specific skill. Additionally, the difference in set scores (i.e., the winner's points minus the loser's points) can help to understand the effect of score balance on performance indicators.

Therefore, the purpose of this study was to analyze technical-tactical performance indicators in terms of the set outcome, game phase, and set score difference. The primary hypothesis of this study is that the performance indicators of the winning team for attacking actions and in the counterattack phase will exceed those of the losing team, irrespective of the set score difference. Furthermore, we predict that the magnitude of the performance indicator difference between the two teams will diminish as the set becomes more balanced. The data presented herein will assist athletes and coaches in comprehending the variables that influence the set outcome in high-level women's beach volleyball.

## 2 Materials and methods

### 2.1 Match samples

The sample included 41 matches with 79 sets and 14,959 game actions [serve: 2,879; serve reception: 2,567; set: 2,176; attack (side-out): 2,324; block: 818; dig: 1,684; set (counterattack): 1,224; and counterattack: 1,287]. Nine sets were excluded because of a failure in the recording. The game actions were collected from two women's 2022 AVP Gold Series tournaments [Atlanta Open (*n* = 18; 43.90%), and Manhattan Beach Open (*n* = 23; 56.10%)] using official broadcasts available on YouTube. The third set of each match was not considered because the number of points to win the set differed from the first and second sets (i.e., 15 points vs. 21 points).

### 2.2 Characterization of the players

The match analyses included 25 beach volleyball teams. All the players who participated in these tournaments were at least “Highly Trained” following McKay et al.'s classification (11). The procedures were approved by a local ethics committee (Human Research Ethics Committee, Health Sciences Centre, Federal University of Paraiba; Opinion no. 4.360.235), and the Declaration of Helsinki was followed.

### 2.3 Variables and instruments

The set outcome and the set score difference were used as independent variables. The set outcome was split into a winner or a loser according to the final set score. The set score difference was classified using a two-step cluster analysis (distance measure: log-likelihood; clustering criterion: Bayesian information criterion). This approach was used to classify the set score difference based on the point difference between the winning and losing sets. Thus, a “large difference” (LD) was characterized by a difference of over 9 points; a “medium difference” (MD) was a difference between 6 and 9 points; and a “small difference” (SD) was a difference between 2 and 5 points. The frequency of each cluster was as follows: LD = 9 sets (11%), MD = 25 sets (32%) and SD = 45 sets (57%).

Concerning the dependent variables, the efficacy score of game actions was classified according to Palao et al. (10). The serve, attack, and block actions were classified into five categories: 0—error (direct point to the opponent), 1—maximum opponent attack options (allowed the opposition the maximum number of options for a counterattack), 2—team limited attack options (allowed the opposition to conduct a limited counterattack), 3—no opponent attack options (does not allow a counterattack formation), and 4—scored points. Furthermore, the serve reception, dig, and set were classified in four categories: 0—error (point to the opponent), 1–no attack option (the opponent regained possession of the ball), 2—limited attack option (allowed perform a limited counterattack), and 3—maximum team attack options (maximum options for a counterattack).

The technical-tactical variables were calculated as follows:

• The points scored during the five game phases [adapted from (6, 8)]. Thus, K0 included points scored by serve; K1 (side-out) included the points scored after receiving the serve and attacking; K2 included points scored by blocking and counterattacking; K3A and K3B were the subsequent points scored by teams in the rally after K1 or K2, respectively. Moreover, the sum of points obtained in counterattacks was calculated (counterattack points = K2 + K3A + K3B).
• The performance coefficient was calculated using Coleman's equations (12). Thus, the equation PC^continuous actions^ = [(1 × “n” efficacy score) + (2 × “n” efficacy score) + (3 × “n” efficacy score 3)/total of actions] was used to calculate continuous actions (i.e., serve reception, set, and dig), and PC^terminal actions^ = [(1 × “n” efficacy score) + (2 × “n” efficacy score) + (3 × “n” efficacy score 3) + (4 × “n” efficacy score 4)]/total of actions was used to calculate terminal actions (i.e., serve, attack, and block).
• Efficiency was calculated using Coleman's equations (12) for attacks and counterattacks separately [Efficiency = (Points − errors) × 100/Attack attempts].

In addition, the number of points in a particular game phase, performance coefficients, and efficiency were classified into “low,” “medium,” and “high” performance categories using two-step cluster analysis [distance measure: log-likelihood; clustering criterion: Bayesian information criterion]. The category with the lowest frequency (≥10) was added to the category closest to it.

### 2.4 Procedure and reliability

The camera was positioned by the American AVP on an elevated plane with a full-court view to record the matches.

Lince® v.1.3 software was utilized for notational analysis (13). The software enabled the input of all categories using the observation tool and the simultaneous visualization of multiple videos within a single window. In addition, it allowed for pausing, rewinding, and reviewing the recorded notations. For further analysis, the notations were exported in a format supported by Microsoft Excel 2016.

The video observation process was conducted by three researchers, each with at least 5 years of experience in beach volleyball. Before data collection, the most experienced researcher led a training phase based on the procedures adopted by Amatria-Jiménez (14). A document outlining the observation criteria for each type of beach volleyball action was provided to the other researchers. Moreover, these criteria were presented in a lecture format, during which potential discrepancies were discussed. Practical training then followed, focusing on the application of the criteria using the Lince software. During this phase, the observers were instructed to watch an action, pause the video, make a notation, and proceed to the next action. They were allowed to rewind the video to re-watch actions when necessary. Moreover, the analyses were conducted independently, without any communication between the researchers’ observations.

Following the training process, intra- and inter-observer reliability were assessed. To this end, the same eight sets (∼10% of the total number of sets) were analyzed twice by all observers, with a 20-day interval between assessments (15). A Kappa coefficient (Ƙ) ≥0.83 for all variables indicated good intra- and inter-observer reliability (16). In addition, to analyze the set of games, the following procedures were adopted: (a) an observer only began analyzing a match after having fully completed the observation of the previous one; (b) all sets of a given game were analyzed by the same observer.

### 2.5 Data analysis

The assumption of normal distribution was supported for all variables, following the recommendations proposed by George and Mallery (17), which consider skewness and kurtosis values between −2 and +2 as acceptable indicators of normality (Supplementary File 1). The data were shown as mean and standard deviation (SD). Moreover, two-way analysis of variance (ANOVA) (between-groups: set outcome, set score difference, and interactions) was used to compare dependent variables (points per game phase, coefficient of performance, and efficiency), and the Bonferroni *post-hoc test* was used for pairwise comparisons. Concerning effect size, the partial eta squared and magnitude were interpreted as follows: small: 0.01; moderate: 0.09; large: 0.25. Moreover, Cohen's “*d*” was used in pairwise comparisons and interpreted as per Hopkins et al.’s (18) recommendation: 0–0.2 (trivial), >0.2–0.6 (small), >0.6–1.2 (moderate), >1.2–2 (large), >2.0–4.0 (very large), and >4 (nearly perfect).

Moreover, binomial univariate and multivariate logistic regressions were performed to verify the relationship between the dependent variable [set result (winner or loser)] and independent variables (performance indicators). The multivariate logistic regression used a forward stepwise method to input variables into the model. Finally, the quality of the model was evaluated using the Hosmer–Lemeshow test, variance inflation factor (VIF), tolerance, and pseudo-R-squared values (*r*^2^). All statistics were calculated using IBM SPSS Statistics for Windows, Version 20.0 (IBM Corp., Armonk, NY, USA), adopting an alpha value of ≤0.05.

## 3 Result

### 3.1 Points scored in the game phases

**Figure 1:**
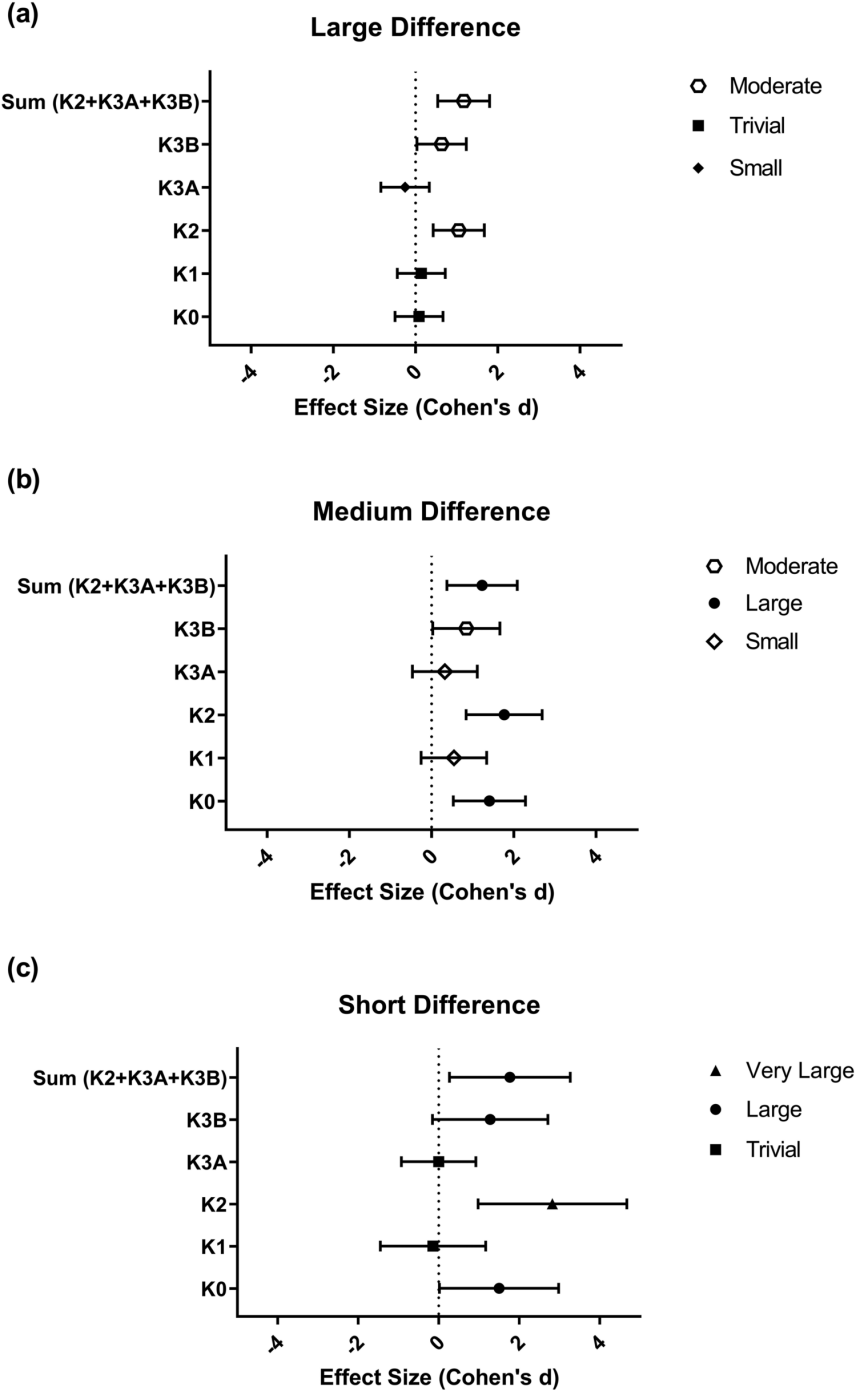
Effect sizes for the differences in points scored across game phases, stratified by set outcome and set score difference. **(a)** Large set score difference; **(b)** medium set score difference; **(c)** short set score difference. Note: A value of *d* > 0 indicates an effect in favor of the winner, while *d* < 0 indicates an effect in favor of the loser. The symbols on the right indicate the magnitude of the effect.

The number of points scored in K0, K2, and ⅀(K2 + K3A + K3B) showed an interaction effect (Table 1). Thus, there was a significant difference between the winner and loser in points scored in K0 when the set was “easy” or “medium,” and there was a difference between the winner and loser in points scored in K2 and ⅀(K2 + K3A + K3B) for all set difficulties. Overall, the winner had an advantage for these performance indicators. Moreover, an outcome effect was observed in K3B [Winner = 1.03 ± 0.156 vs. Loser = 0.264 ± 0.143; *F*_(1.00, 152.00)_ = 21.465; *p* ≤ 0.001; *η_ρ_*^2^ = 0.124]. Concerning effect size in pairwise analysis, K2 was the best performance indicator for a “LD” set score difference; K0, K2, and ⅀points^(K2^ ^+^ ^K3A^ ^+^ ^K3B)^ were the best performance indicators for a “MD” set score difference; and K2 and ⅀points^(K2^ ^+^ ^K3A^ ^+^ ^K3B)^ were the best performance indicators for a “SD” set score difference (Figure 1).

**Table 1:** Comparison of points scored in different game phases stratified by set outcome and set score difference.

Game phase	Winner						Loser						Two-way ANOVA (Interaction)
	LD (*n* = 9)		MD (*n* = 25)		SD (*n* = 45)		LD (*n* = 9)		MD (*n* = 25)		SD (*n* = 45)		
	Mean	SD(±)	Mean	SD(±)	Mean	SD(±)	Mean	SD(±)	Mean	SD(±)	Mean	SD(±)	
K0	1.22^a^	0.83	1.72^a^	1.24	0.98	1.01	0.22	0.44	0.36	0.57	0.89	1.11	*F*_(2.0, 152.0)_ = 6.939; *p* < 0.001; *η_ρ_*^2^ = 0.084*
K1	4.78	1.72	7.24	2.13	8.27	1.95	5.00	1.41	6.16	1.82	8.00	1.85	*F*_(2.0, 152.0)_ = 1.080; *p* = 0.342; *η_ρ_*^2^= 0.014
K2	5.22^a^	2.28	4.28^a^	2.13	3.40^a^	1.63	0.44	0.73	1.32	1.03	1.93	1.12	*F*_(2.0, 152.0)_ = 10.516; *p* < 0.001; *η_ρ_*^2^ = 0.122*
K3A	1.11	0.93	1.72	1.21	1.62	1.09	1.11	0.78	1.36	0.99	1.93	1.36	*F*_(2.0, 152.0)_ = 1.356; *p* = 0.261; *η_ρ_*^2^ = 0.018
K3B^b^	1.11	1.05	1.08	1.26	0.91	0.95	0.11	0.33	0.28	0.46	0.40	0.62	*F*_(2.0, 152.0)_ = 0.880; *p* = 0.417; *η_ρ_*^2^ = 0.011
⅀^(K2^ ^+^ ^K3A^ ^+^ ^K3B)^	7.44^a^	3.09	7.08^a^	2.60	5.93^a^	2.08	2.96	1.67	4.27	1.91	3.56	1.96	*F*_(2.0, 152.0)_ = 10.302; *p* < 0.001; *η_ρ_*^2^ = 0.119*

SD, standard deviation; *η_ρ_*^2^, partial eta squared; LD, large difference; MD, medium difference; SD, short difference.

^a^ Statistically significant difference (*p* ≤ 0.05) between the winning and losing teams in terms of the set score difference (LD, MD, SD).

^b^ Statistically significant difference (*p* ≤ 0.05) between the winner vs. loser teams.

* *p* ≤ 0.05.

### 3.2 Performance coefficients

**Figure 2:**
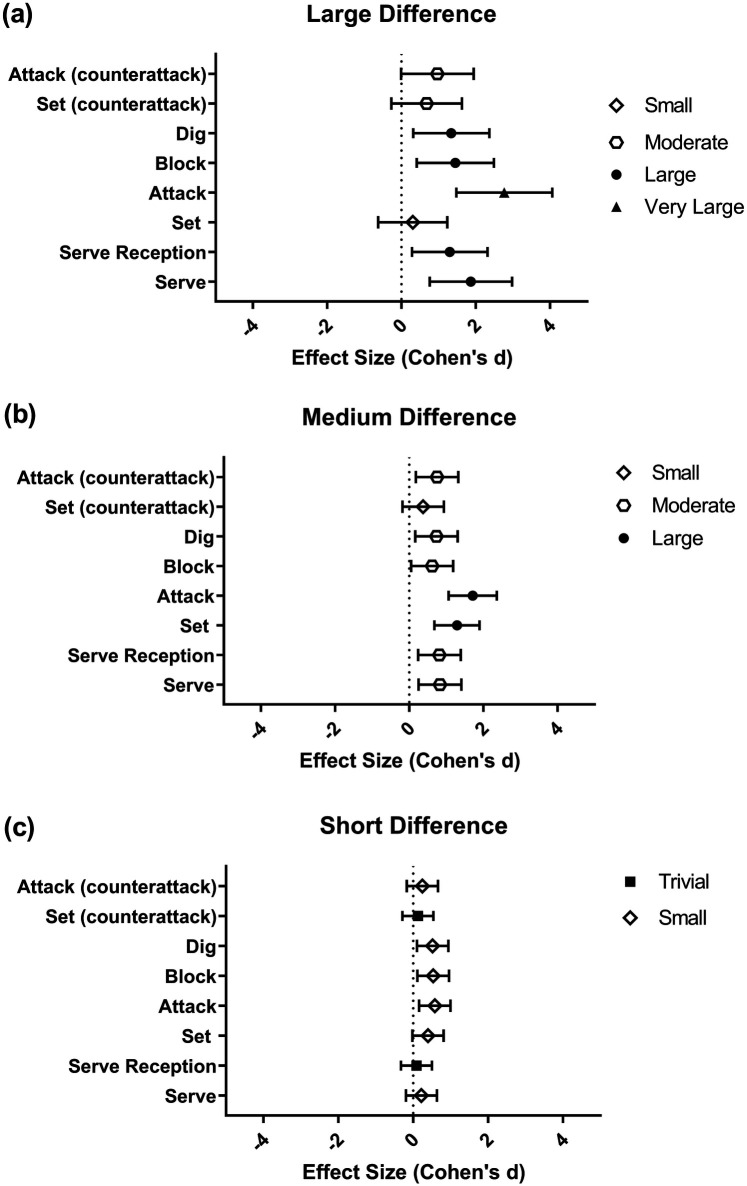
Effect sizes for the differences in performance indicators between winners and losers across different set score differences. **(a)** Large set score difference; **(b)** medium set score difference; **(c)** short set score difference. Note: A value of *d* > 0 indicates an effect in favor of the winner, while *d* < 0 indicates an effect in favor of the loser. The symbols on the right represent the magnitude of the effect.

The serve, serve reception, set, and attack performance coefficients showed an interaction effect (Table 2). Thus, serve and serve reception showed a significant difference for the “LD” and “MD” set score differences, set only showed a significant difference for the “MD” set score difference, and attack showed a significant difference for all set score differences. Moreover, an outcome effect was observed for block [Winner = 1.81 ± 0.70 vs. Loser = 1.32 ± 0.84; *F*_(1.00, 152.00)_ = 17.094; *p* ≤ 0.001; *η_ρ_*^2^ = 0.102], dig [Winner 2.07 ± 0.08 vs. 0.40 ± 0.09; *F*_(1.00, 152.00)_ = 21.230; *p* ≤ 0.001; *η_ρ_*^2^ = 0.123, set (counterattack) [Winner = 2.03 ± 0.40 vs. Loser = 1.73 ± 0.45; *F*_(1.00, 152.00)_ = 21.230; *p* ≤ 0.001; *η_ρ_*^2^ = 0.123], and attack (counterattack) [Winner = 2.77 ± 0.54 vs. Loser = 2.42 ± 0.80; *F*_(1.00, 152.00)_ = 12.389; *p* = 0.001; *η_ρ_*^2^ = 0.072]. Regarding effect size, the pairwise analysis showed that attack was the best performance indicator in sets with the “LD” set score difference, with set and attack the best for the “MD” set score difference, and attack, block, and dig the best for the “SD” set score difference (Figure 2).

**Table 2:** Comparison of performance coefficients and efficiency stratified by set outcome and set score difference.

Performance indicators	Winner						Loser						Two-way ANOVA^(Interaction)^
	LD (*n* = 9)		MD (*n* = 25)		SD (*n* = 45)		LD (*n* = 9)		MD (*n* = 25)		SD (*n* = 45)		
	Mean	SD(±)	Mean	SD(±)	Mean	SD(±)	Mean	SD(±)	Mean	SD(±)	Mean	SD(±)	
PC serve	1.81^a^	0.21	1.64^a^	0.39	1.47	0.35	1.27	0.35	1.32	0.39	1.39	0.37	*F*_(2.00, 152.00)_ = 3.798; *p* = 0.025; *η_ρ_*^2^ = 0.048
PC serve reception	2.48^a^	0.37	2.43^a^	0.38	2.35	0.35	2.09	0.20	2.15	0.30	2.32	0.31	*F*_(2.00, 152.00)_ = 3.533; *p* = 0.032; *η_ρ_*^2^ = 0.044
PC set	2.68	0.39	2.81^a^	0.27	2.78	0.31	2.56	0.40	2.39	0.37	2.65	0.35	*F*_(2.00, 152.00)_ = 3.293; *p* = 0.040; *η_ρ_*^2^ = 0.042
PC attack	3.09^a^	0.55	2.97^a^	0.43	2.84^a^	0.41	1.96	0.17	2.26	0.40	2.60	0.42	*F*_(2.00, 152.00)_ = 10.975; *p* < 0.001; *η_ρ_*^2^ = 0.126
PC block^b^	1.94	0.75	1.84	0.71	1.77	0.70	0.83	0.78	1.33	0.92	1.33	0.92	*F*_(2.00, 151.00)_ = 2.361; *p* = 0.098; *η_ρ_*^2^ = 0.030
PC dig^b^	2.16	0.36	2.06	0.49	1.98	0.35	1.53	0.55	1.74	0.37	1.76	0.48	*F*_(2.00, 152.00)_ = 1.723; *p* = 0.182; *η_ρ_*^2^ = 0.022
PC set (CT)^b^	2.25	0.26	2.48	0.43	2.37	0.36	1.89	0.70	2.31	0.48	2.32	0.41	*F*_(2.00, 152.00)_ = 1.070; *p* = 0.346; *η_ρ_*^2^ = 0.014
PC attack (CT)^b^	2.86	0.45	2.88	0.46	2.69	0.60	2.15	0.94	2.32	0.95	2.53	0.69	*F*_(2.00, 152.00)_ = 2.071; *p* = 0.130; *η_ρ_*^2^ = 0.027
EFF attack	49.81^a^	19.30	53.18^a^	18.32	45.19	18.89	04.80	14.50	21.77	15.23	32.89	14.92	*F*_(2.00, 152.00)_ = 9.730; *p* < 0.001; *η_ρ_*^2^ = 0.113*
EFF attack (CA)^b^	53.06	16.20	50.20	21.58	40.78	22.88	21.98	33.90	26.98	37.78	31.69	29.27	*F*_(2.00, 152.00)_ = 1.780; *p* = 0.172; *η_ρ_*^2^ = 0.023

PC, performance coefficient; EFF, efficiency; CA, counterattack; SD, standard deviation; *η_ρ_*^2^, partial eta squared; LD, large difference; MD, medium difference; SD, short difference.

^a^ Statistically significant difference (*p* ≤ 0.05) between the winning and losing teams in terms of the set score difference (LD, MD, SD).

^b^ Statistically significant difference (*p* ≤ 0.05) between the winner vs. loser teams.

* *p* ≤ 0.05.

### 3.3 Efficiency

**Figure 3:**
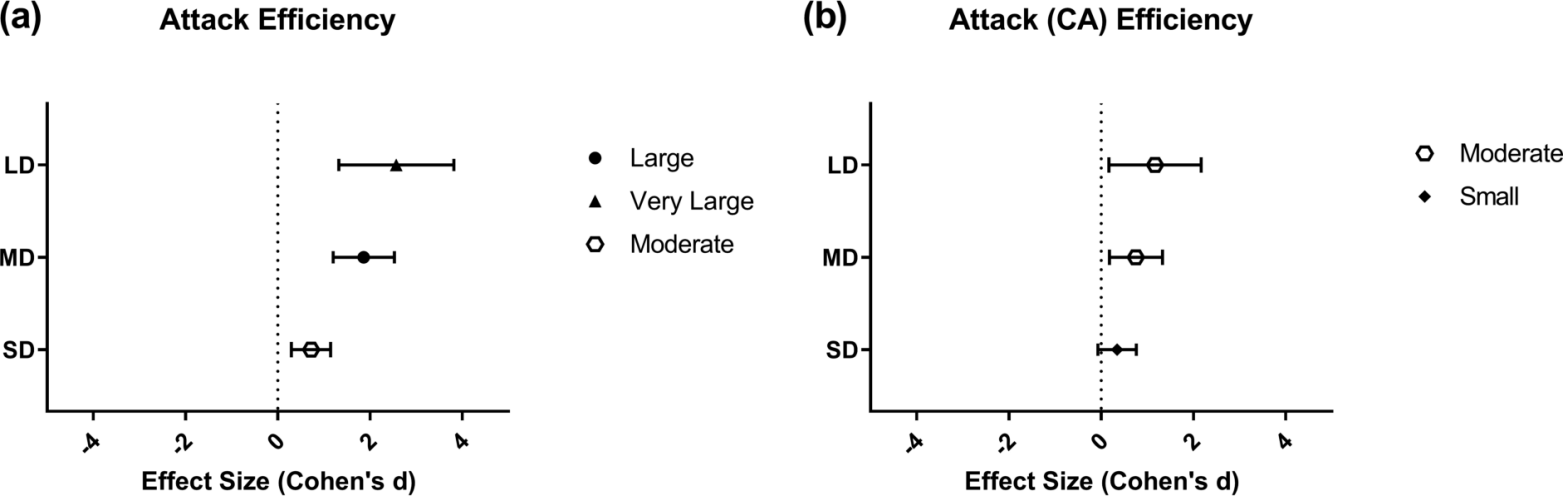
Effect sizes for the differences in attack efficiency **(a)** and counterattack efficiency **(b)** between winners and losers across different set score differences. Note: LD, large difference; MD, medium difference; SD, short difference. A value of *d* > 0 indicates an effect in favor of the winner, while *d* < 0 indicates an effect in favor of the loser. The symbols on the right indicate the magnitude of the effect.

The attack efficiency showed an interaction effect (Table 2). Thus, the attack efficiency had a significant difference for all set score differences. Moreover, an outcome effect was observed for attack (counterattack) efficiency [Winner: 45.16 ± 22.18 vs. Loser: 29.09 ± 32.43; *F*_(2–152)_ = 14.991; *p* ≤ 0.001; *η_ρ_*^2^ = 0.090]. Concerning effect size, the pairwise analysis showed that attack efficiency had the largest effect size (Figure 3).

### 3.4 Univariate and multivariate logistic regression between the set outcome and performance indicators

The categorized performance indicators were used in the logistic regression (Table 3). For the attack and attack (counterattack) performance coefficients, the “medium” and “high” categories were combined for the analysis due to the low frequency of the “high” classification.

**Table 3:** Performance indicator categories.

Performance indicator	Performance categories		
	Low	Medium	High
K0	0	1	≥2
K1	≤5	6–7	≥8
K2	≤1	2–3	≥4
K3A	≤1	2	≥3
K3B	0	1	≥2
⅀(K2 + K3A + K3B)	≤3	4–6	≥7
PC serve	≤1.40	1.41–1.80	≥1.85
PC server reception	≤2.21	2.23–2.70	≥2.75
PC set	≤2.26	2.31–2.76	≥2.77
PC attack	≤2.56	2.63–3.25	≥3.27
PC block	≤1.33	1.40–2.22	≥2.40
PC dig	≤1.63	1.66–2.42	≥2.66
PC set (CA)	≤2.30	2.33–2.77	≥2.80
PC attack (CA)	≤2.40	2.42–3.30	≥3.40
EFF attack	≤18.18	18.75–46.15	≥46.66
EFF counterattack	≤0.00	10.00–44.40	≥45.50

PC, performance coefficient; EFF, efficiency; CA, counterattack.

Categorized by distance measure: log-likelihood; clustering criterion: Bayesian information criterion method.

In the univariate analyses, a significant relationship was observed between set outcome and K0, K2, K3B, and ⅀ points^(K2^ ^+^ ^K3A^ ^+^ ^K3B)^; the serve, serve reception, set, attack, block, dig, and counterattack performance coefficients; and attack and counterattack efficiencies (more details can be found in Supplementary File 2). K2 and ⅀points^(K2^ ^+^ ^K3A^ ^+^ ^K3B)^ had the highest г^2^ values (0.46 and 0.44, respectively).

In the multivariate analysis, Model 1 included the serve, serve reception, set, attack, block, dig, and counterattack performance coefficients, and the attack and counterattack efficiencies. A significant relationship was observed between set outcome and the serve, set, block, and dig performance coefficients, and the attack and counterattack efficiencies, regardless of the performance classification (Table 4). Moreover, “high” counterattack efficiency, “high” attack efficiency, and “high” block and dig performance coefficients were the best performance indicators, increasing the probability of winning the set by 26.207, 18.490, 15.033, and 13.811 times, respectively, compared to the “low” performance classification. Regarding model quality, there was no multicollinearity [VIFmean = 1.07 (±0.06); tolerance: 0.93 (±0.05)], the expected frequencies were not different from those observed (Hosmer–Lemeshow test: *p* = 0.684), 60% of the variability in set outcome was explained by the model, and the accuracy of the predictions was 82.91%.

**Table 4:** Logistic regression between the set outcome and performance indicators—Model 1.

Set outcome^a^	*β*	SE	Odds ratio	Sig.
Performance indicator^b^				
PC serve	—	—	—	0.007*
Medium	1.822	0.651	6.186	0.005*
High	1.159	0.574	3.186	0.044
PC set	—	—	—	0.043*
Medium	0.236	0.805	1.267	0.769
High	1.408	0.700	4.088	0.044*
PC block	—	—	—	0.003*
Medium	0.840	0.515	2.316	0.103
High	2.710	0.804	15.033	<0.001*
PC dig	—	—	—	0.006*
Medium	1.646	0.565	5.187	0.004*
High	2.625	1.046	13.811	0.012*
EFF attack	—	—	—	<0.001*
Medium	1.216	0.717	3.375	0.090
High	3.266	0.810	26.207	<0.001*
EFF counterattack	—	—	—	<0.001*
Medium	1.721	0.744	5.590	0.021*
High	2.917	0.809	18.490	<0.001*

PC, performance coefficient; EFF, efficiency; CA, counterattack.

Hosmer–Lemeshow test: *p* = 0.684; *r*^2^ = 0.605; VIF^mean^ = 1.07 (±0.06); tolerance: 0.93 (±0.05); model's accuracy = 82.91%.

^a^ Winner was used as the reference category.

^b^ Low was used as the reference category.

* *p* ≤ 0.05.

In Model 2, the points scored in the game phases variable was added. A significant relationship was observed between the set outcome and points scored in K0, K2, and K3B, and the set performance coefficient was classified as “high” (Table 5). Moreover, a significant trend was observed for the “medium + high” attack performance coefficient (*p* = 0.058). It is noteworthy that “high” performances in K2 and K3B were the main performance indicators, increasing the likelihood of winning the set by 604.90 and 181.37 times, respectively, compared to a “low” performance. In relation to model quality, there was no multicollinearity [VIF^mean^ = 1.54 (±0.06); tolerance: 0.73 (±0.27)], the expected frequencies were not different from those observed (Hosmer–Lemeshow test: *p* = 0.735), 78% of the variability in set outcome was explained by the model, and the accuracy of the predictions was 90.50%.

**Table 5:** Logistic regression between the set outcome and performance indicators—Model 2.

Set outcome^a^	*β*	SE	Odds ratio	Sig.
Performance indicator^b^				
K0	—	—	—	0.039*
Medium	1.205	0.724	3.336	0.096
High	1.932	0.774	6.907	0.013*
K2	—	—	—	<0.001
Medium	1.328	0.763	3.772	0.082
High	6.405	1.363	604.904	<0.001*
K3B	—	—	—	<0.001*
Medium	1.149	0.682	3.155	0.092
High	5.201	1.240	181.371	0.000*
PC Set	—	—	—	0.061
Medium	1.366	1.152	3.919	0.236
High	2.377	1.084	10.771	0.028*
PC attack (medium + high)	2.256	1.188	9.544	0.058
EFF attack	—	—	—	0.016*
Medium	−0.968	1.172	0.380	0.409
High	1.195	1.410	3.304	0.397
Constant	−7.188	1.648	0.001	<0.001

PC, performance coefficient; EFF, efficiency.

Hosmer–Lemeshow test: *p* = 0.735; *r*^2^ = 0.789; VIF^mean^ = 1.54 (±0.65); tolerance: 0.73 (±0.27); model's accuracy = 90.50%.

^a^ Winner was used as the reference category.

^b^ Low was used as the reference category.

* *p* ≤ 0.05.

## 4 Discussion

The purpose of this study was to analyze technical-tactical performance indicators in terms of the set outcome, set score difference, and game phase. The winners scored the most points in all game phases, had a higher performance coefficients and efficiency than the losers, independent of the set score difference. However, the set score difference seemed to influence the difference between the winner and the loser. Thus, a significant difference in points scored in game phases was observed for points scored in K0 for the “LD” and “MD” set score differences, and for points scored in K2 and ⅀points^(K2^ ^+^ ^K2+^ ^K3B)^ for set score difference overall. Furthermore, the winners had better serve, serve reception, and attack performance coefficients in sets with an “LD” set score difference; serve reception and attack performance coefficients in sets with an “MD” set score difference; and attack performance coefficient in sets with an “SD” set score difference than the losers. Additionally, winners showed superior performance coefficients in blocking, setting (counterattack), and attack efficiency (counterattack), independently of set score difference. Regarding the magnitude of differences, both the effect sizes and the number of key performance indicators distinguishing winners from losers tended to decrease as the set score difference decreased, suggesting that, in closely contested sets, fewer indicators determine the outcome. Moreover, the logistic regression analyses showed a positive relationship between winning a set and attack efficiency, attack (counterattack) efficiency, dig and block performance coefficients (see Model 1), and points scored in K2 and K3B (see Model 2). In general terms, the data seem to confirm the initial hypotheses.

The serve is a player's first opportunity to score points in a rally or at least impair the organization of their opponent's attack. Previously, Medeiros et al. (6) observed that the winning male teams scored more points than the losers with their serve, which corroborates our data. However, the aim of the serve reception phase is to control the ball to organize an effective attack. In sets with “easy” and “medium” difficulties, points in K0 and the serve and serve reception performance coefficients seem to differentiate winners and losers, but this did not happen in “hard” sets. Thus, in easier sets, the winning team was able to serve more effectively and neutralize the opponent's serve through a strong serve-reception performance. Moreover, high-quality reception facilitates setting and allows the player to apply the appropriate technique according to the tactical demands (8).

In this sense, although the number of points scored in K0 was ∼2–3 points per set, which is a small contribution to scoring 21 points to win the set, and serve reception and setting are actions that do not score points, these actions interfere with or are fundamental support for an attack. Our data showed that attack performance (K1) is important for victory since the attack performance coefficient and efficiency differentiated the outcome and/or were associated with winning a set, as an attack not only allows a player to score a point but also harms the opponent's counterattack. In a previous study, Medeiros et al. (6) found in a study on male players of various levels that points scored in K1 had a small effect on winning the set, but the performance coefficient and errors seemed to have a moderate to large effect. In women, this effect appears to be similar; however, we found that in sets with a “LD” set score difference, the attack performance coefficient and efficiency determined the winner. In other words, it is unlikely that a team will win a set without a superior performance in K1. Moreover, using absolute point scores should be avoided when evaluating athletes’ performance in this game phase.

Concerning the counterattack phases (K2, K3A, and K3B), points scored in K2 and K3B seem to be the main performance indicators in high-level women's beach volleyball. The effect of ⅀points^(K2^ ^+^ ^K3A^ ^+^ ^K3B)^ on the outcome was probably leveraged by the performance in K2 and K3B; thus, this performance indicator does not present additional advantages in match analysis. Our data partially corroborate what was previously observed in male athletes (6), as although K2 was an important indicator of victory, K3B was favorable to losers. These data suggest that the adoption of parameters related to technical-tactical performance should be interpreted according to gender. Although some performance indicators are common to both men and women, there may be some differences. When analyzing the counterattack actions specifically, a block, a dig, and an attack (counterattack) were the main indicators of victory. However, blocking and defending operate similarly to serve and serve reception. The importance of these actions is not based on the points obtained, but on creating opportunities for a counterattack. Previously, it was identified that winners are superior in points scored by blocking (9); however, this results in a limited number of points (i.e., 1–2 per set), reinforcing the idea of blocking being a secondary indicator of victory in a set.

In general, this data can be used as a benchmark for the performance of athletes competing at a high level. Based on the knowledge of the factors that lead to winning a set in women’s high-performance beach volleyball, some strategies can be adopted in training and competitions. Concerning one’s serve, using either a power jump or floating jump serve and aiming for the central and line zones seems to improve performance (19). Furthermore, the speed of the ball has been found to be related to scoring a point (20); however, the players and coaches must keep in mind that serving at a very high speed increases the chances of error (20). Therefore, an effective serving strategy to score points is to direct the ball between opposing players or toward the boundary lines at a speed of at least 12–16 m s^−1^ (19, 20). Moreover, these strategies can impair the serve reception, increase the performance coefficient, help a player score a point in another game phase, and consequently increase the chance of winning the set.

Concerning serve reception, training should provide opportunities for players to receive float serves or jump float serves because they are the types predominantly used by female players (21). In addition, serve reception and setting can be trained together because they naturally occur in sequence in a game, and this will allow players to adapt to their teammate's serve-reception behavior. Regarding blocking and digging, these actions are strongly related, and strategies are usually adopted before the attack action. However, parsing visual information and decision-making are fundamental (22). Therefore, these aspects need to be present in training. Moreover, the failure of an attack during K1 usually leads a player to modify their subsequent attack [i.e., shot or smash (23)] and athletes need to be aware of this. Finally, during attacking actions, female players perform shots and smashes with similar frequency (21). Therefore, training should not focus solely on the execution of the attacking action but also emphasize the importance of perceiving the opponent's positioning. This ensures that even technically perfect attacks are not executed out of context of the tactical demands of the match.

Regarding the presentation of data to players, it is recommended that the following performance indicators be employed: the efficiency of the attack and counterattack; the dig and block performance coefficients; and points scored in K2 and K3B. These metrics are fundamental for winning a set and avoiding redundancy of information, in line with the idea of key performance indicators (24). Finally, some limitations of this study need to be acknowledged. The match analyses were from two AVP Gold Series events, which bring together the best athletes in the AVP rankings. Therefore, this data should be extrapolated with caution to grassroots and lower-level athletes. In addition, the stages of the competition (i.e., group stage, semi-finals, etc.) were not taken into account; in future studies, this could be included as a moderating factor. Another important point is that we did not consider the moments of the match (i.e., start of the game, set points, etc.), which may in the future provide important insights into the crucial moment of victory. Finally, future studies could consider incorporating qualitative inferential analysis approaches (e.g., polar coordinate analysis) to enhance the understanding of performance indicators in beach volleyball.

## 5 Conclusion

In conclusion, attack efficiency was an important performance indicator. However, it is important to highlight that the main indicators of performance were derived from the points scored and actions performed during the counterattack phases, especially K2 and K3B. In addition, the set score difference reflects the balance of the set (i.e., the smaller the score difference, the smaller the difference in performance indicators between the winner and the loser of the set). Therefore, these parameters can be used to guide training programs (e.g., increased training volume focusing on a dig during a counterattack) and evaluate team performance (e.g., superiority over the opponent).

## Data Availability

The raw data supporting the conclusions of this article will be made available by the authors, without undue reservation.
