# Supplementary material for: Comparison and association of performance indicators according to set outcome and set score difference in AVP women's beach volleyball
Source: Front Sports Act Living. 2025 Aug 11;7:1584173. doi: 10.3389/fspor.2025.1584173 (PMC12376430; doi:10.3389/fspor.2025.1584173)
Supplement: Supplementary file 1 [file Supplementaryfile1.docx]

Supplementary File 1. Skewness and kurtosis indicators and normality classification of distribution

| **Variables** | **Skewness** | **Kurtosis** | **Classification of distribution normality^a^** |
| --- | --- | --- | --- |
| EFF – Attack | -0,184 | -0,23 | Normal |
| EFF – Attack (CA) | -0,249 | 0,091 | Normal |
| PC - Serve | 0,219 | -1,446 | Normal |
| PC - Serve Reception | 0,62 | -0,656 | Normal |
| PC - Set | -0,899 | -0,592 | Normal |
| PC - Attack | -0,032 | -0,486 | Normal |
| PC - Block | -0,115 | 0,034 | Normal |
| PC - Digd | -0,181 | 1,061 | Normal |
| PC - Set (CT) | -0,151 | 0,363 | Normal |
| PC - Attack (CT) | -0,302 | 0,065 | Normal |
| K0 | 1,117 | 0,935 | Normal |
| K1 | -0,163 | -0,647 | Normal |
| K2 | 0,737 | 0,385 | Normal |
| K3A | 0,659 | 0,531 | Normal |
| K3B | 1,463 | 1,917 | Normal |
| **⅀^(K2+K3A+K3B)^** | 0,469 | -0,17 | Normal |

^a^According to George and Mallery’s (2010) criteria: skewness and kurtosis values between -2 and +2 indicate acceptable normality.
