# Supplementary material for: Comparison and association of performance indicators according to set outcome and set score difference in AVP women's beach volleyball
Source: Front Sports Act Living. 2025 Aug 11;7:1584173. doi: 10.3389/fspor.2025.1584173 (PMC12376430; doi:10.3389/fspor.2025.1584173)
Supplement: Supplementary file 2 [file Supplementaryfile2.docx]

Supplementary File 2. Univariate logistic regression between set outcome (winner/loser - dependent) and performance indicators (independent)

| **Set Outcome^a^** | **B** | **S.E.** | **Wald** | **df** | **Sig.** | **Odds Ration** | **r^2^** |
| --- | --- | --- | --- | --- | --- | --- | --- |
| **Performance indicators^b^** |  |  |  |  |  |  |  |
| **K0** |  |  | **14.238** | **2** | **0.001** |  | 0.126 |
| Medium | 0.588 | 0.386 | 2.316 | 1 | 0.128 | 1.800 |  |
| High | 1.624 | 0.431 | 14.224 | 1 | 0.000 | 5.073 |  |
| **K1** |  |  | **2.075** | **2** | **0.354** |  | 0.018 |
| Medium | 0.076 | 0.443 | 0.029 | 1 | 0.865 | 1.078 |  |
| High | 0.499 | 0.398 | 1.572 | 1 | 0.210 | 1.647 |  |
| **K2** |  |  | **35.177** | **2** | **0.000** |  | 0.469 |
| Medium | 0.834 | 0.469 | 3.158 | 1 | 0.076 | 2.302 |  |
| High | 4.169 | 0.714 | 34.079 | 1 | 0.000 | 64.625 |  |
| **K3A** |  |  | **0.386** | **2** | **0.825** |  | 0.003 |
| Medium | 0.076 | 0.371 | 0.042 | 1 | 0.838 | 1.079 |  |
| High | 0.258 | 0.416 | 0.386 | 1 | 0.534 | 1.295 |  |
| **K3B** |  |  | **16.773** | **2** | **0.000** |  | 0.178 |
| Medium | 0.822 | 0.371 | 4.911 | 1 | 0.027 | 2.275 |  |
| High | 2.506 | 0.656 | 14.597 | 1 | 0.000 | 12.250 |  |
| **⅀ points^(K2 + K3A+K3B)^** |  |  | 39.597 | 2 | 0.000 |  | 0.447 |
| Medium | 2.273 | 0.533 | 18.189 | 1 | 0.000 | 9.710 |  |
| High | 4.206 | 0.670 | 39.407 | 1 | 0.000 | 67.080 |  |
| **PC Serve** |  |  | **14.195** | **2** | **0.001** |  | 0.120 |
| Medium | 1.341 | 0.433 | 9.573 | 1 | 0.002 | 3.821 |  |
| High | 1.191 | 0.396 | 9.028 | 1 | 0.003 | 3.290 |  |
| **PC Serve reception** |  |  | **6.275** | **2** | **0.043** |  | 0.054 |
| Medium | 0.668 | 0.347 | 3.699 | 1 | 0.054 | 1.951 |  |
| High | 1.110 | 0.521 | 4.539 | 1 | 0.033 | 3.034 |  |
| **PC Set** |  |  | **14.950** | **2** | **0.001** |  | 0.129 |
| Medium | 0.570 | 0.512 | 1.240 | 1 | 0.265 | 1.768 |  |
| High | 1.615 | 0.473 | 11.648 | 1 | 0.001 | 5.026 |  |
| **PC Attack** |  |  |  |  |  |  | 0.233 |
| Medium+High | 1.920 | 0.373 | 26.449 | 1 | 0.000 | 6.819 |  |
| **PC Block** |  |  | **6.922** | **2** | **0.031** |  | 0.060 |
| Medium | 0.743 | 0.352 | 4.458 | 1 | 0.035 | 2.103 |  |
| High | 1.195 | 0.533 | 5.029 | 1 | 0.025 | 3.304 |  |
| **PC Dig** |  |  | **9.523** | **2** | **0.009** |  | 0.088 |
| Medium | 1.023 | 0.407 | 6.314 | 1 | 0.012 | 2.781 |  |
| High | 2.246 | 0.869 | 6.690 | 1 | 0.010 | 9.455 |  |
| **PC Set (CA)** |  |  | **3.099** | **2** | **0.212** |  | 0.026 |
| Medium | 0.670 | 0.383 | 3.058 | 1 | 0.080 | 1.954 |  |
| High | 0.306 | 0.413 | 0.547 | 1 | 0.459 | 1.358 |  |
| **PC Attack (CA)** |  |  |  |  |  |  | 0.097 |
| Medium+High | 1.158 | 0.343 | 11.393 | 1 | 0.001 | 3.183 |  |
| **EFF Attack** |  |  | **29.627** | **2** | **0.000** |  | 0.270 |
| Medium | 0.793 | 0.495 | 2.568 | 1 | 0.109 | 2.211 |  |
| High | 2.617 | 0.537 | 23.781 | 1 | 0.000 | 13.690 |  |
| **EFF Attack (CA)** |  |  | **15.156** | **2** | **0.001** |  | 0.148 |
| Medium | 1.501 | 0.595 | 6.362 | 1 | 0.012 | 4.487 |  |
| High | 2.303 | 0.612 | 14.138 | 1 | 0.000 | 10.000 |  |

^a^ = Winner as reference category; ^b^ = low classification as reference category. CA = counterattack; * p ≤ 0.05
